# Neural Encoding of Auditory Statistics

**DOI:** 10.1523/JNEUROSCI.1887-20.2021

**Published:** 2021-08-04

**Authors:** Benjamin Skerritt-Davis, Mounya Elhilali

**Affiliations:** Johns Hopkins University, Baltimore, Maryland 21218

**Keywords:** auditory perception, computational modeling, EEG, multifeature integration, psychophysics, statistical inference

## Abstract

The human brain extracts statistical regularities embedded in real-world scenes to sift through the complexity stemming from changing dynamics and entwined uncertainty along multiple perceptual dimensions (e.g., pitch, timbre, location). While there is evidence that sensory dynamics along different auditory dimensions are tracked independently by separate cortical networks, how these statistics are integrated to give rise to unified objects remains unknown, particularly in dynamic scenes that lack conspicuous coupling between features. Using tone sequences with stochastic regularities along spectral and spatial dimensions, this study examines behavioral and electrophysiological responses from human listeners (male and female) to changing statistics in auditory sequences and uses a computational model of predictive Bayesian inference to formulate multiple hypotheses for statistical integration across features. Neural responses reveal multiplexed brain responses reflecting both local statistics along individual features in frontocentral networks, together with global (object-level) processing in centroparietal networks. Independent tracking of local surprisal along each acoustic feature reveals linear modulation of neural responses, while global melody-level statistics follow a nonlinear integration of statistical beliefs across features to guide perception. Near identical results are obtained in separate experiments along spectral and spatial acoustic dimensions, suggesting a common mechanism for statistical inference in the brain. Potential variations in statistical integration strategies and memory deployment shed light on individual variability between listeners in terms of behavioral efficacy and fidelity of neural encoding of stochastic change in acoustic sequences.

**SIGNIFICANCE STATEMENT** The world around us is complex and ever changing: in everyday listening, sound sources evolve along multiple dimensions, such as pitch, timbre, and spatial location, and they exhibit emergent statistical properties that change over time. In the face of this complexity, the brain builds an internal representation of the external world by collecting statistics from the sensory input along multiple dimensions. Using a Bayesian predictive inference model, this work considers alternative hypotheses for how statistics are combined across sensory dimensions. Behavioral and neural responses from human listeners show the brain multiplexes two representations, where local statistics along each feature linearly affect neural responses, and global statistics nonlinearly combine statistical beliefs across dimensions to shape perception of stochastic auditory sequences.

## Introduction

In everyday environments, the brain sifts through a plethora of sensory inputs, tracking pertinent information along multiple dimensions despite the persistent uncertainty in real-world scenes. Inferring statistical structure in complex environments is a hallmark of perception that facilitates robust representation of sensory objects as they evolve along different perceptual dimensions (or features, used interchangeably). Evidence of statistical inference has been documented in audition (Creel et al., 2004; Agus et al., 2010; Pearce et al., 2010; Krishnan et al., 2019); vision (Fiser and Aslin, 2002; Brady et al., 2009) and olfaction (Degel, 2001), as well as across sensory modalities (Conway and Christiansen, 2005; Frost et al., 2015), showing that it underlies the encoding of sensory surroundings in memory.

Predictive coding offers a framework to explain how these mnemonic representations of the past guide interpretation of future sensory inputs. The theory posits that the brain builds an internal model of the external world to make probabilistic predictions of future events (Friston, 2005; Heilbron and Chait, 2018; Seriès and Seitz, 2013). In audition, the oddball paradigm has been used extensively to demonstrate the ability of the brain to track predictive structures, or regularities, along various auditory dimensions such as pitch, loudness, duration, timbre, and spatial location (Schröger and Wolff, 1996; Takegata et al., 2005; Pakarinen et al., 2007; Vuust et al., 2016). Many neurophysiology studies have shown that the brain makes predictions along multiple features simultaneously (Takegata et al., 1999; Paavilainen et al., 2001; Caclin et al., 2006; Du et al., 2011; Pieszek et al., 2013). However, these studies do not give any indication of how these independent predictions are combined at later stages of processing to give rise to integrated object-level percepts. It is clear through behavioral studies (and everyday experience) that listeners integrate across features to represent sound sources wholly as objects (Melara and Marks, 1990; Thompson and Sinclair, 1993; Dyson and Ishfaq, 2008; Shinn-Cunningham et al., 2008; Chernyshev et al., 2016). What is not clear is the manner in which independently tracked sensory dimensions are joined into a unified statistical representation that reflects the complexity and nondeterministic nature of natural listening scenarios.

To address the limitations of quasi-predictable regularities often used in previous studies, we focus on the perception of stochastic regularities that exist in the continuum between perfectly predictable and completely random. We use stimuli exhibiting random fractal structure (also known as 1/*f* or power-law noise) along multiple features, both spectral and spatial. Random fractals occur in natural sounds, including music and speech (Pickover and Khorasani, 1986; Attias and Schreiner, 1997; Geffen et al., 2011; Levitin et al., 2012), and previous work has shown the brain is sensitive to these types of structures (Schmuckler and Gilden, 1993; Garcia-Lazaro et al., 2006; Overath et al., 2007; Maniscalco et al., 2018; Skerritt-Davis and Elhilali, 2018). Using a change detection paradigm, we task listeners with detecting changes in the entropy of sound sequences along multiple features. With this paradigm, we probe the ability of the brain to abstract statistical properties from complex sound sequences in a manner that has not been addressed by previous work. Importantly, the statistical structure of the sequences used in this study carry no particular coupling or correlation across features hence restricting the ability of the brain to leverage this correspondence, which is in line with previously reported feature fusion mechanisms observed within and between visual, somatosensory, vestibular, and auditory sensory modalities (Treisman and Gelade, 1980; Angelaki et al., 2009; Fetsch et al., 2010; Parise et al., 2012; Ernst et al., 2013).

The use of an experimental paradigm involving uncertainty raises the specific challenge of interpretation of responses to each stochastic stimulus, as changes in underlying statistics need not align with behavioral and neural responses to the instantiations of these statistics. This complexity is further compounded in multidimensional feature spaces, begging the question of how the brain deals with this uncertainty, especially with dynamic sensory inputs that lack a priori dependencies across dimensions.

The current study develops a computational model to guide our analysis through simulation and to make inferences about the underlying computational mechanisms behind multidimensional predictive coding in the brain. This model offers the opportunity to ask the following targeted questions. Which statistics are tracked along each feature? When does integration across features occur? Are features combined linearly or through some other function? The model is used to formulate alternative hypotheses addressing these questions and to compare them by simulating listener responses in the behavioral paradigm. In addition, we use the output of the model as an anchor for time-locking analysis of neural responses, combating the temporal uncertainty that invariably creeps into the analysis of stochastic responses to stochastic stimuli.

## Materials and Methods

### Experimental design and statistical analyses

We conducted four experiments to probe the mechanisms behind predictive processing along multiple dimensions in auditory perception, as follows: two psychophysics experiments (Experiments SP and TP) and two similarly structured electroencephalography (EEG) experiments (Experiments nSP and nTP, with “n” denoting neural). Listeners were asked to detect changes in the statistical properties of a sequence of complex sounds varying along the following two perceptual features: in Experiments SP and nSP, stimuli varied in spatial location (S) and pitch (P), as denoted by the naming convention; in Experiments TP and nTP, stimuli varied in timbre (T) and pitch (P).

#### Participants

In Experiment SP, 16 participants (8 females) were recruited from the general population (mean age, 25.1 years); 1 participant was excluded from further analysis because their task performance was near chance (*d^′^* < 0.05). In Experiment TP, 18 participants (12 females) were recruited (mean age, 21.5 years); 3 participants were excluded because of chance performance. In Experiment nSP, 20 participants (9 females) were recruited (mean age, 23.4 years); 2 participants were excluded because of chance performance. In Experiment nTP, 22 participants (13 females) were recruited (mean age, 22.5 years); 4 participants were excluded because of chance performance. Sample sizes were estimated based on similar experiments previously reported (Skerritt-Davis and Elhilali, 2018).

All participants reported no history of hearing loss or neurologic problems. Participants gave informed consent before the experiment and were paid for their participation. All experimental procedures were approved by the Johns Hopkins University institutional review board.

#### Stimuli

Stimuli in all experiments were melodies composed of a sequence of complex tones varying along two perceptual features. Stimuli in Experiments SP and nSP varied in pitch and spatial location; stimuli in Experiments TP and nTP varied in pitch and timbre. To simulate the variability present in natural sounds, each feature followed the contour of a random fractal at different levels of entropy or randomness.

Random fractals are stochastic processes with spectrum inversely proportional to frequency with log-slope β (i.e., 1/*f*^β^), where β parameterizes the entropy of the sequence. Fractals at three levels of entropy were used as seed sequences to generate the stimuli: low (β = 2.5), mid (β = 2), and high (β = 0, white noise). In all experiments, stimuli began with both features at lower entropy, and halfway through the melody, one or both features increased to high entropy. Stimuli with decreasing entropy were not used in this work for two reasons: (1) to keep the duration of the multifeature paradigm within a single experimental session; and (2) our previous work shows no difference in behavioral responses based on direction of entropy change (Skerritt-Davis and Elhilali, 2018). We chose to use increasing entropy because it affords the brain more of a chance to initiate tracking of statistics jointly across features at the onset of the stimulus.

**Figure 1.**
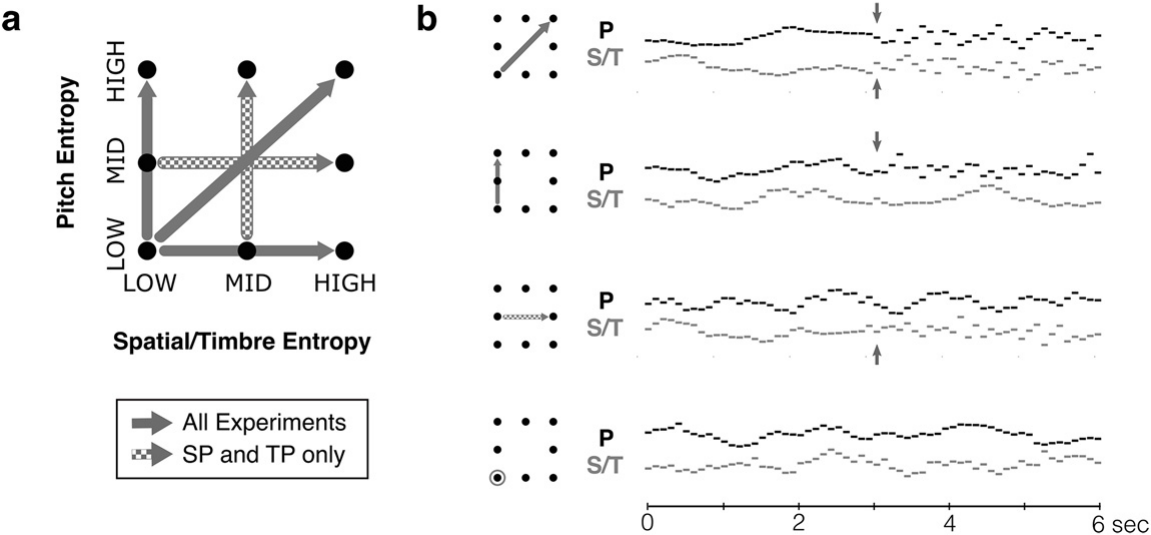
Multifeature stochastic stimuli. ***a***, Stimuli were melodies comprised of tones varying according to stochastic regularities along two features simultaneously: Pitch and spatial location (in Experiments SP and nSP); or pitch and timbre (in Experiment TP and nTP). At the midpoint of the melody, one or both features increased in entropy (nondiagonal and diagonal arrows, respectively), while the nonchanging feature remained at low entropy. For psychophysics experiments (SP and TP), the nonchanging feature could also have mid-level entropy (checkered arrows). ***b***, Four example stimulus sequences with condition indicated by small schematic on left. Arrows indicate change in each feature, when present. The bottom example is a control trial with no change.

In the psychophysics experiments (SP and TP), for stimulus conditions with a single feature changing, the nonchanging feature could have either low or mid entropy. In the EEG experiments (nSP and nTP), the nonchanging feature always had low entropy. Control conditions contained stimuli with no entropy change in either feature. See Figure 1 for an illustration of the different stimulus conditions in each experiment.

Each complex tone in the melody sequence was synthesized from a harmonic stack of sinusoids with frequencies at integer multiples of the fundamental frequency, then high- and low-pass filtered at the same cutoff frequency using fourth-order Butterworth filters. Pitch was manipulated through the fundamental frequency of the complex tone, and timbre was manipulated through the cutoff frequencies of the high- and low-pass filters (i.e., the spectral centroid; Allen et al., 2017). Spatial location was simulated by convolving the resulting tone with interpolated head-related impulse functions for the left and right ear at the desired azimuthal position (Algazi et al., 2001). Seed fractals were generated independently for each feature and each stimulus, standardized (i.e., zero mean and unit variance), and then mapped to feature space, as follows:

$$\begin{array}{l} F_{0}[t]=350*2^{3x[t]/12}\\ S[t]=15y[t]\\ T[t]=1200*2^{3z[t]/12}, \end{array}$$

where *F*_0_[*t*], *S*[*t*], and *T*[*t*] are pitch (fundamental frequency in hertz), spatial location (azimuth in degrees), and timbre (spectral centroid in hertz) sequences indexed by time *t*. *x*[*t*], *y*[*t*], and *z*[*t*] are their respective seed fractals. Fundamental frequency ranged from 208 to 589 Hz, spatial location ranged from –45^∘^ to 45^∘^ azimuth at 0^∘^ elevation, and spectral centroid (timbre) ranged from 714 to 2018 Hz.

**Figure 2.**
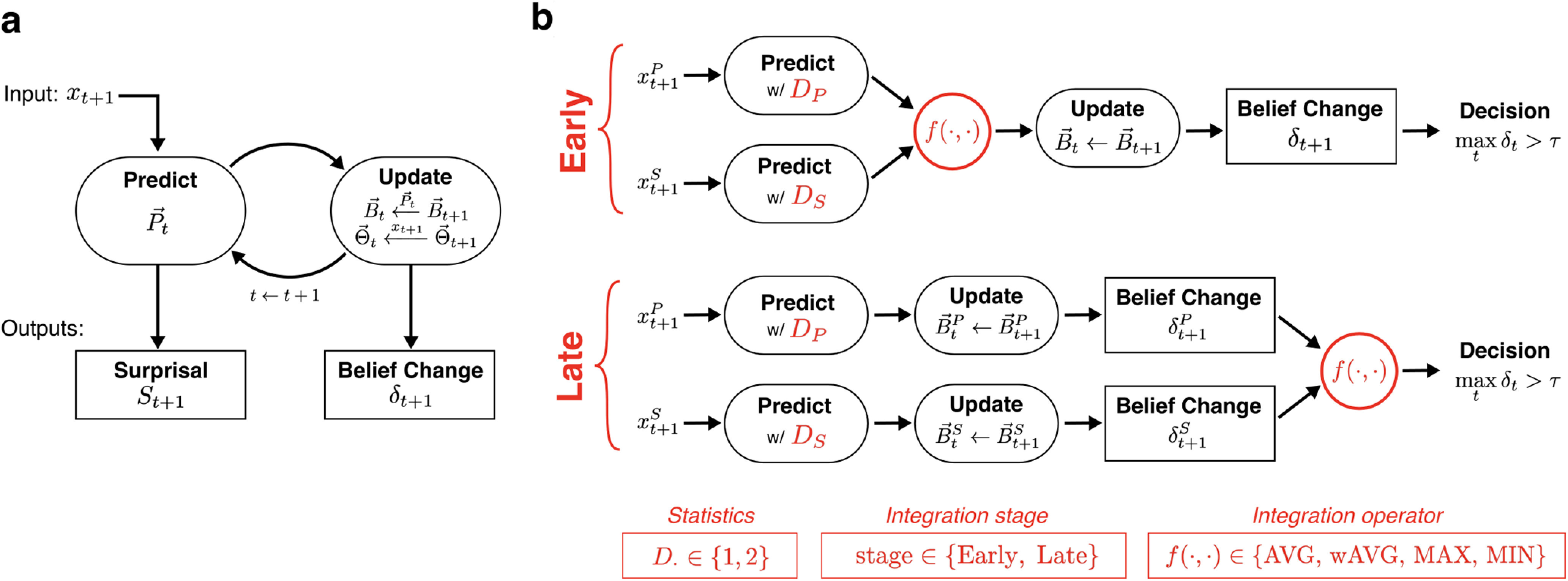
Model schematic. ***a***, Building blocks of the model for predictive processing along a single dimension. ***b***, Illustration of potential variants of the model for statistical inference along multiple dimensions. Red indicates aspects of the model that differed by variant: statistics collected along each dimension (*D* ∈ {1,2}), early-stage versus late-stage integration, and the operator used in integration (MAX, MIN, AVG, wAVG). Summary of model variants are in red boxes at bottom.

**Figure 3.**
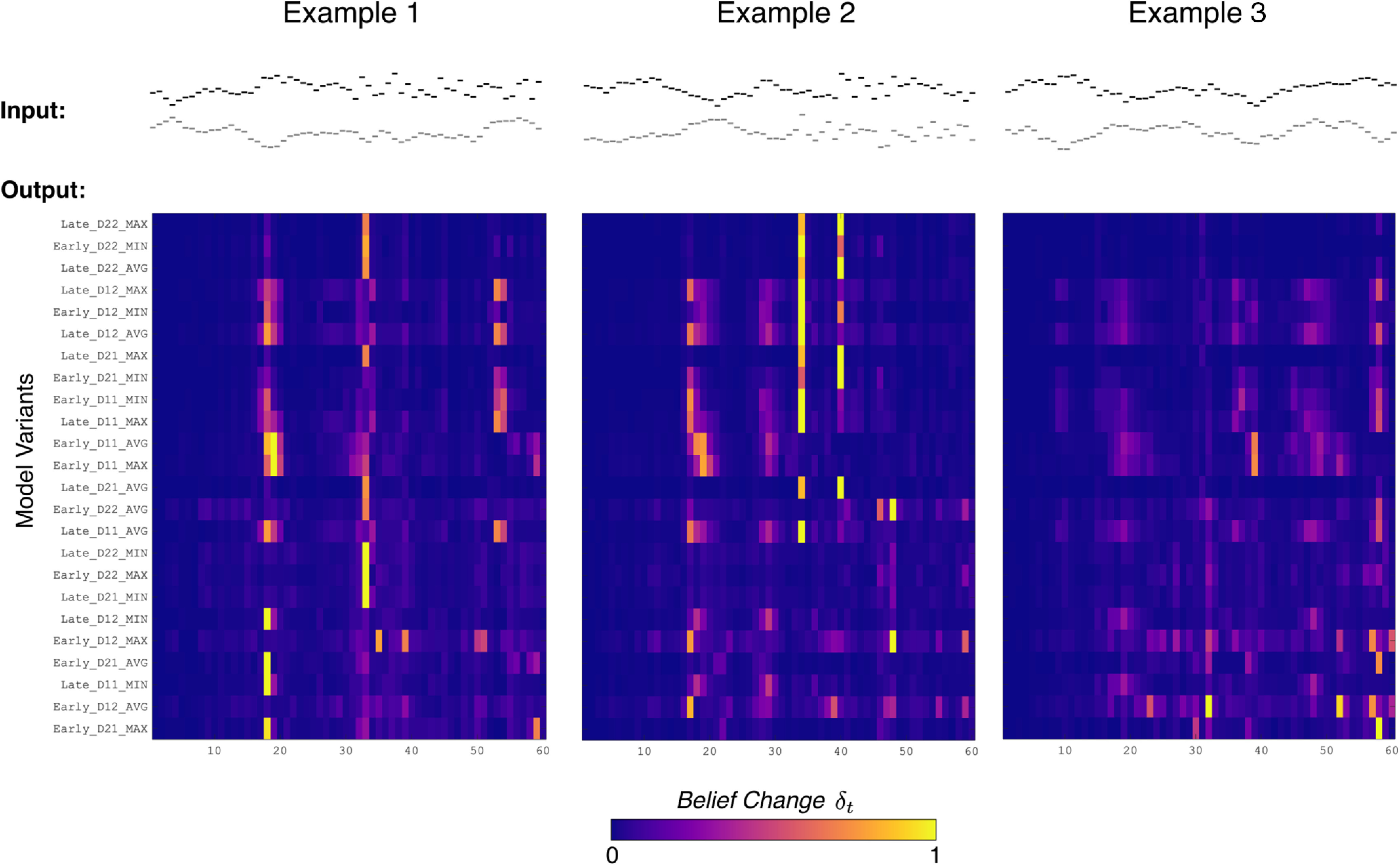
Belief change response from model variants for three example stimuli. Belief change response from model (δ*_t_*) shown over time alongside example stimuli (top). Horizontal axes show time in observations. Each row is a model variant, ordered by the average fit loss across subjects found in Experiment nSP (i.e., top row has minimum loss). wAVG variants were excluded in this figure. Examples are from the following stimulus conditions: (1) single-feature change; (2) both-feature change; and (3) no change (control).

**Figure 4.**
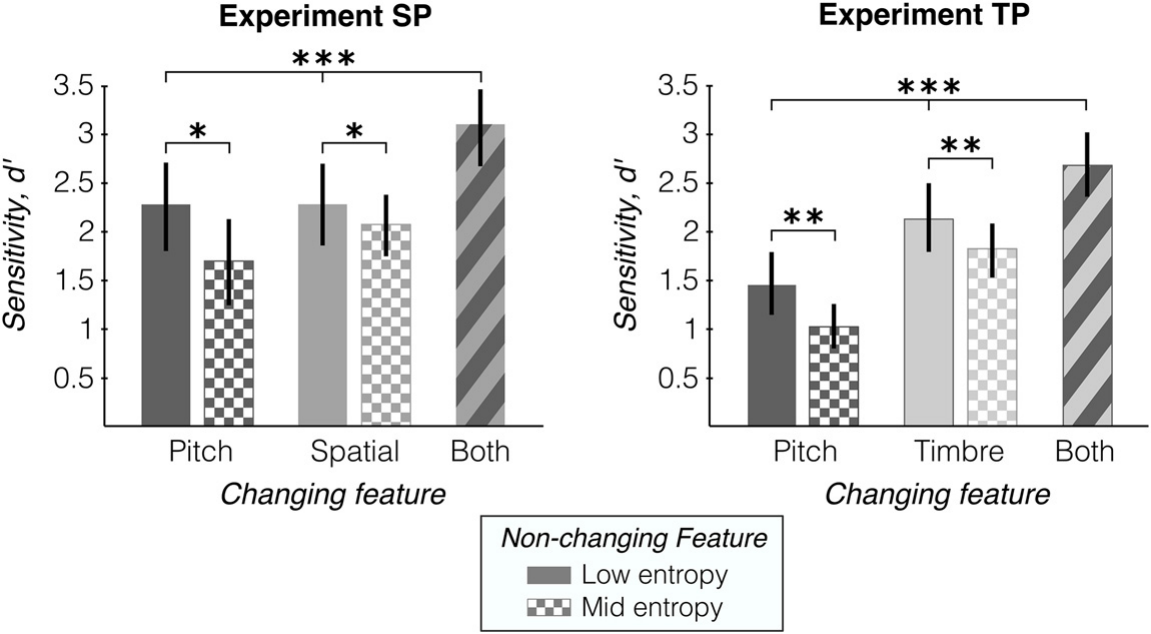
Behavioral results for Experiments SP and TP. Average change detection performance (*d^′^*) is shown by changing feature (abscissa) and entropy of nonchanging feature (fill pattern). Error bars indicate 95% bootstrap confidence interval across subjects (*N* = 15 for both experiments).

**Figure 5.**
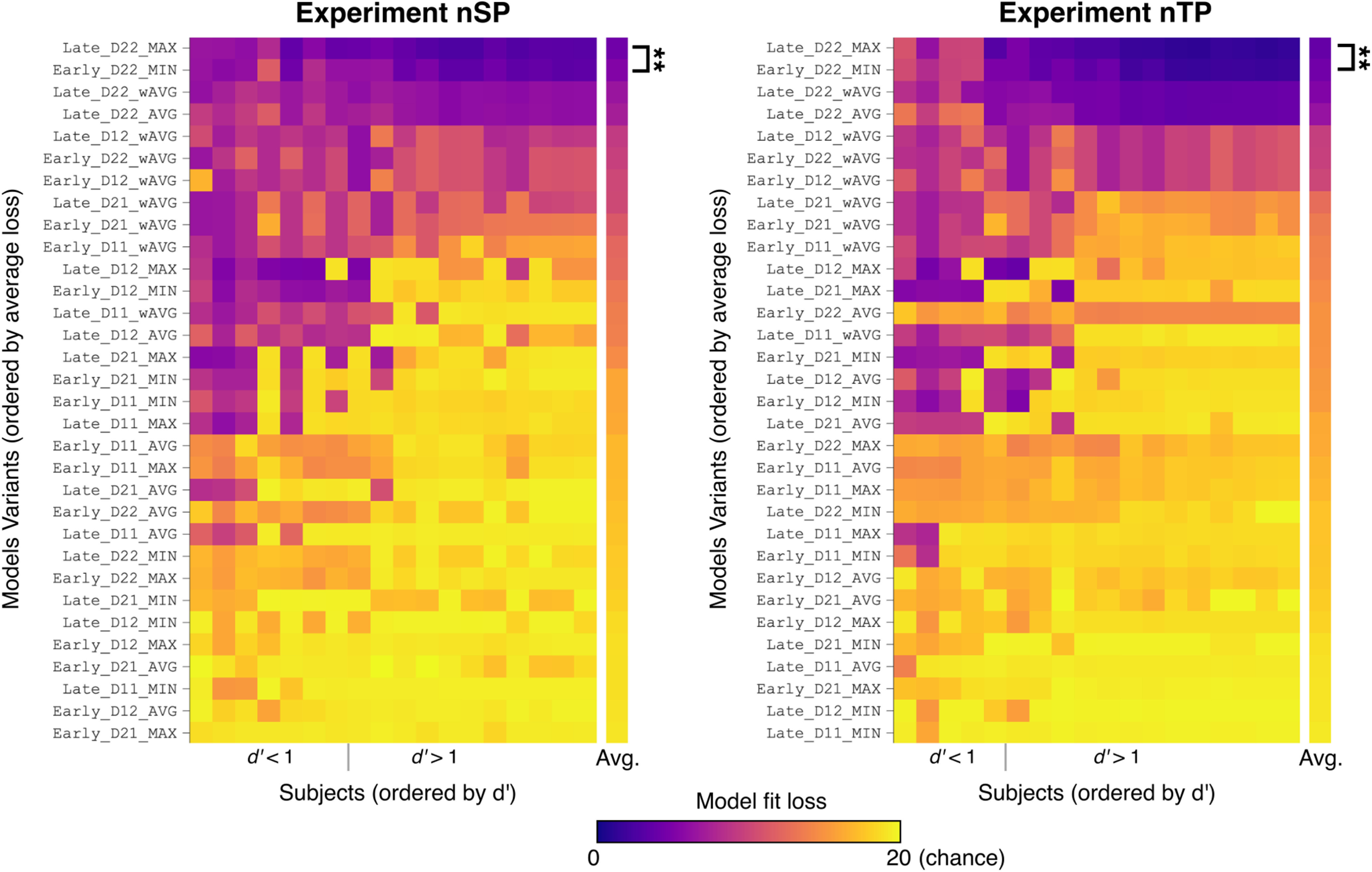
Model comparison in Experiments nSP and nTP. Each model variant was fit to individual subjects, and the resulting loss is displayed by color. Each row is a model variant (ordered by average loss), and each column is a subject (ordered by *d^′^*). Model names to the left of each image indicate integration stage, statistics (*D*) collected for each feature, and integration operator. The two best models, Late_D22_MAX and Early_D22_MAX, were compared using a *t* test (*N* = 18 in both experiments).

**Figure 6.**
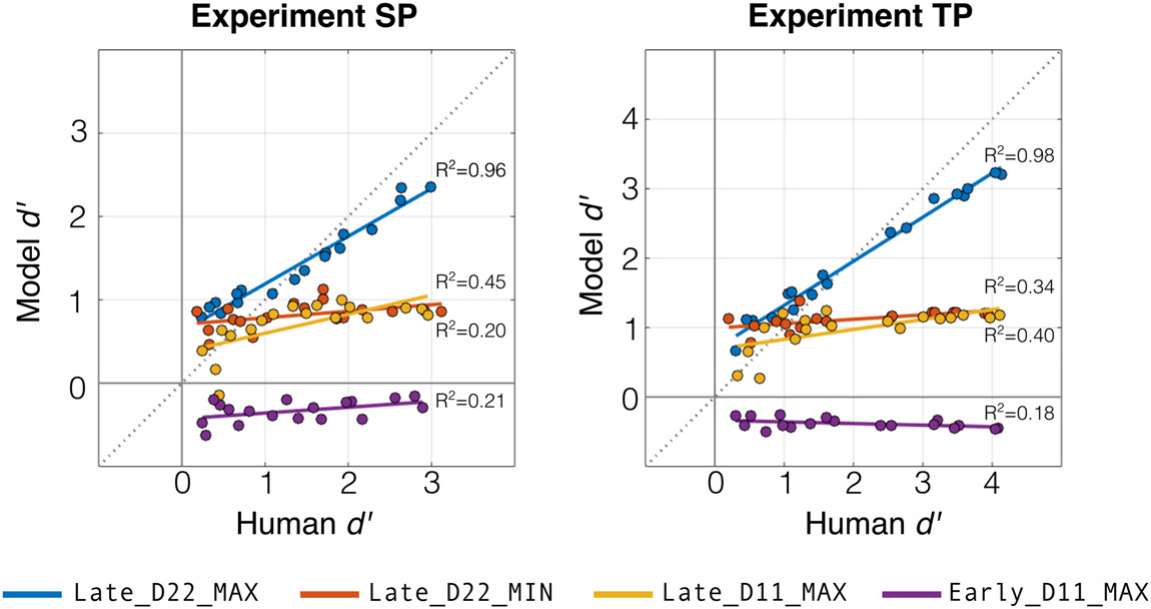
Cross-validation comparison between fitted model and listener behavior. Task performance (*d^′^*) on a held-out test partition is plotted for four model variants (denoted by color) against listener performance in Experiments SP and TP. For each experiment, models were fit to each subject using a random partition of experiment trials, and evaluation results on the test partition were averaged over 10 cross-validation iterations. The dotted line on the diagonal indicates an ideal model, where the fitted performance replicates human behavior.

**Figure 7.**
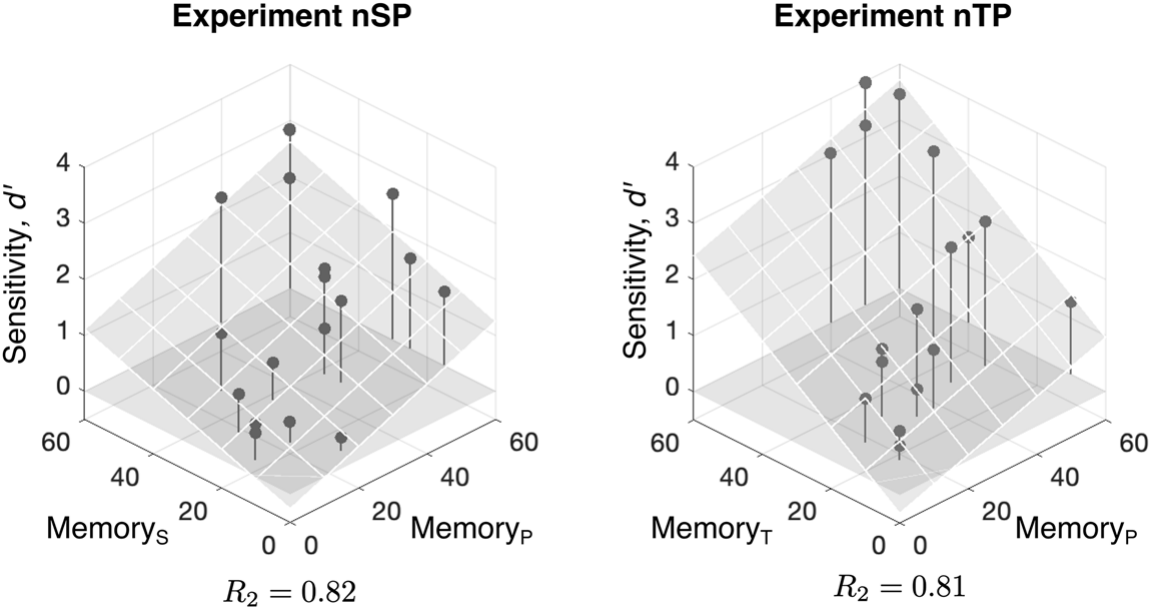
Memory parameters of the Late_D22_MAX model fit to individual subjects in Experiments nSP and nTP. Fitted memory parameters plotted against overall detection performance *d^′^*, along with multiple linear regression fit (*R*^2^ is at the bottom of each plot). Observation noise parameters (data not shown) did not have a significant correlation with *d^′^*.

**Figure 8.**
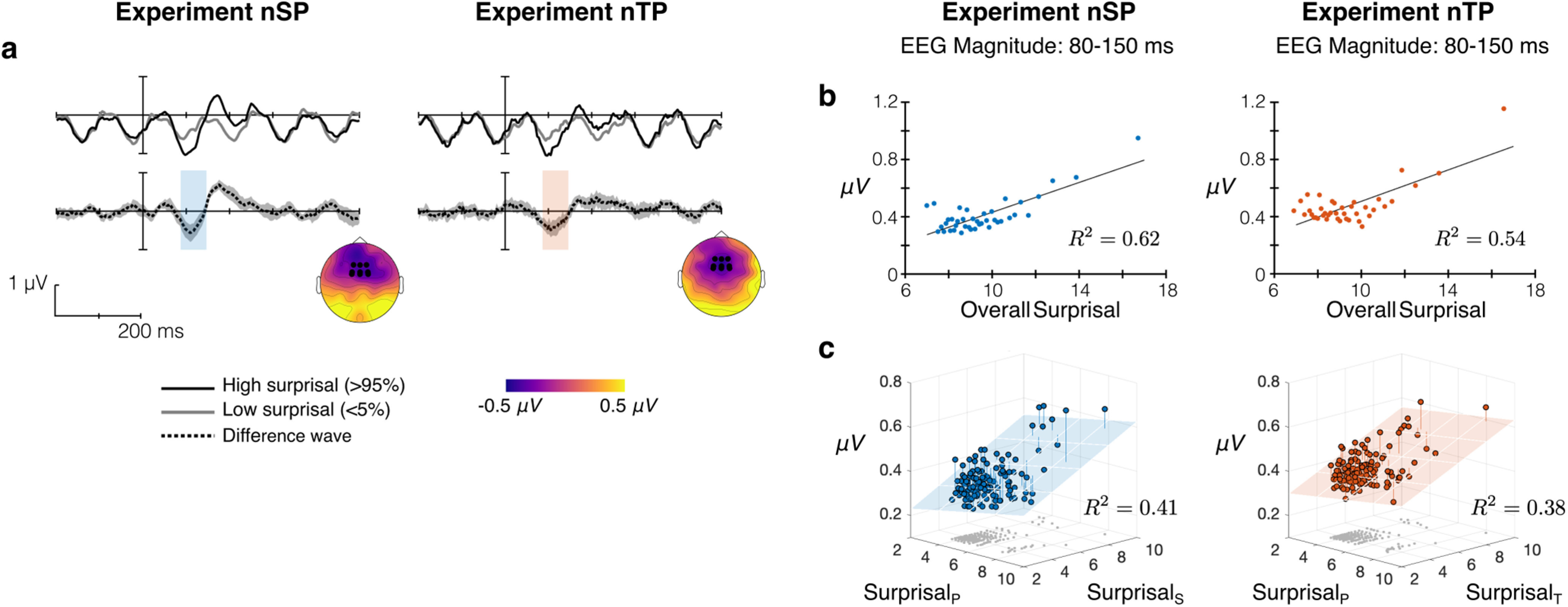
Surprisal response in Experiments nSP and nTP. ***a***, Oddball-like analysis contrasting neural response to high-surprisal tones (top 5%) with response to low-surprisal tones (bottom 5%), where overall surprisal is summed across features (e.g., $S_{t}^{P}+S_{t}^{T}$). Difference wave (high-low) shows 95% confidence interval across subjects. ***b***, EEG magnitude (80–150 ms) in subaverages of tone epochs binned by overall surprisal (abscissa). *R*^2^ from linear regression. ***c***, EEG magnitude (80–150 ms) binned by feature-specific surprisal in both features (horizontal axes). Gray points on horizontal axis show the position of each point in surprisal space. *R*^2^ is from multiple linear regression.

In Experiments SP and TP, melody stimuli were composed of 60 complex tones, each 100 ms in duration with 20 ms onset/offset ramps presented isochronously at a rate of 10 Hz. Two hundred stimuli were generated, 25 for each condition (5 change, 3 no-change). In Experiments nSP and nTP, melody stimuli were composed of 60 complex tones, each 100 ms in duration with 20 ms onset/offset ramps presented isochronously at a rate of 8.6 Hz. Two hundred stimuli were generated, 50 for each condition (3 change, 1 no-change). Audio examples of multifeature stimuli are included in Fig. 1-1, Fig. 2-1, Fig. 3-1, Fig. 4-1, Fig. 5-1, Fig. 6-1, Fig. 7-1, Fig. 8-1.

#### Procedure

Stimuli were presented in randomized order; thus, listeners did not know a priori which feature was informative for the task. The experiment contained four blocks with self-paced breaks between blocks. During each trial, participants were instructed to listen for a change in the melody. After the melody finished, participants responded via keyboard whether or not they heard a change. Immediate feedback was given after each response.

Listeners were not given explicit instructions about what to listen for, learning the task implicitly in a training block before testing. Incorrect responses in the training block resulted in the same stimulus being replayed with feedback (including, in the case of missed detections, a visual indication of change during playback).

Stimuli were synthesized on-the-fly at a 44.1 kHz sampling rate and presented at a comfortable listening level using PsychToolbox (psychtoolbox.org) and custom scripts in MATLAB (MathWorks). Participants were seated in an anechoic chamber in front of the presentation screen.

In Experiments SP and TP, stimuli were presented via over-ear headphones (model HD 595, Sennheiser), and participants responded via keyboard. The experiment duration was ∼50 min. In Experiments nSP and nTP, stimuli were presented via in-ear headphones (model ER-2, Etymotic) and participants responded via response box. Additionally, before each melody trial, a fixation cross appeared on the screen to reduce eye movement during EEG acquisition. The experiment duration, including EEG setup, was ∼120 min.

#### Statistical analyses

In all experiments, the effects of stimulus condition on behavioral responses were assessed using repeated-measures ANOVA to control for differences across subjects, with factors reported for each experiment in the main text in the Results section, and significant effects seen in Figure 4. *Post hoc* analyses using *t* tests were used to determine the extent of observed effects for individual conditions.

### Computational model

We used the dynamic regularity extraction (D-REX) model to investigate the computational underpinnings of multidimensional predictive processing (Skerritt-Davis and Elhilali, 2018). The model builds robust sequential predictions by exploiting statistical properties of sounds; thus, it is a potential computational solution for how the brain tracks natural sound sources that are not perfectly predictable but rather have predictive structures that change over time.

Initially designed to build statistical predictions along a single dimension, we used the D-REX model as a framework to generate potential implementations in multiple dimensions. We specified a set of model variants a priori, each representing a different hypothesis for how the brain tracks statistics along multiple dimensions. For each model variant, we then simulated model responses to the same stimuli in Experiments nSP and nTP, selecting model parameters that maximized agreement between model and individual listener behavioral responses. The best model was then used to interpret neural responses.

In this section, we give a brief overview of the principles behind the D-REX model and how it was used to formulate hypotheses for the computational mechanisms behind predictive processing of multifeature sounds.

#### Statistical inference along a single dimension.

The D-REX model builds sequential predictions of the next input *x_t_*_+1_, given all previously observed inputs $x_{1},x_{2},...,x_{t}$. In the present study, the input ${\left\{x_{t}\right\}}_{t\in {\mathbb{Z}}^{+}}$ is a sequence of pitches, spatial locations, or spectral centroids (timbre). This sensory input is assumed to be successively drawn from a multivariate Gaussian distribution with unknown parameters, as this structure encompasses sequentially unfolding sounds in a wide range of natural and experimental phenomena (Winkler et al., 1990; Attias and Schreiner, 1997; Nelken et al., 1999; Daikhin and Ahissar, 2012; Garrido et al., 2013; Skerritt-Davis and Elhilali, 2019). Over time, the model collects sufficient statistics $\hat{\theta }$ from observed inputs to estimate the unknown distribution parameters (Murphy, 2007).

If the generating distribution were stationary, this would yield the following prediction equation:

(1) $$\mathbb{P}(x_{t+1}|x_{1:t})=\mathbb{P}(x_{t+1}|\hat{\theta }(x_{1:t})),$$

where $\hat{\theta }(x_{1:t})=\{{\hat{\mu }}_{D}(x_{1:t}),{\hat{\Sigma }}_{D}(x_{1:t})\}$ are the sufficient statistics of the underlying distribution collected over the observed inputs: the sample mean and sample covariance, respectively. The subscript *D* denotes the dimensionality of these statistical estimates, and, equivalently, the distributional assumption in the generative model of the input sequence {*x_t_*}. For *D* = 1, the observations are assumed to be temporally independent, and the model uses a univariate Gaussian, collecting mean and variance from the observed inputs. For *D* = 2, successive observations are assumed to be temporally dependent, and the model additionally collects covariances between adjacent inputs. In this manner, we use the model to test for different statistics collected by the brain.

While Equation 1 suffices if the underlying distribution is stationary, modeling the dynamics of real-world environments requires relaxing this assumption. The D-REX model assumes the unknown parameters of the underlying distribution change at unknown times, and observations before and after each change are independently distributed. Consequently, the context window of past observations relevant for the current prediction depends on when the most recent change occurred. To build predictions robust to these unknown dynamics, the model entertains multiple potential context windows $\vec{C}=\{c_{i}\}$, with a corresponding set of statistical estimates at time *t*, ${\vec{\Theta }}_{t}=\{{\hat{\theta }}_{i,t}\}$; i∈{1,...,M}. Each ${\hat{\theta }}_{i,t}$ is sufficient statistics collected over context *c_i_*, and *M* is the total number of context hypotheses (representing a maximal memory capacity for the model).

When a new input *x_t_*_+1_ is observed, the model produces a predictive probability of this input for each context hypothesis, as follows:

(2) $$\begin{array}{l} {\vec{P}}_{t}=\{p_{i,t}\},i\in \{1,...,M\}\\ p_{i,t}=\mathbb{P}(x_{t+1}|{\hat{\theta }}_{i,t}), \end{array}$$

where *p_i,t_* is the context-specific predictive probability of *x_t_*_+1_, given the statistics estimated over the *i*th context hypothesis. Alongside these predictive probabilities, the model maintains a set of beliefs for each context hypothesis, as follows:

(3) $$\begin{array}{l} {\vec{B}}_{t}=\{b_{i,t}\},i\in \{1,...,M\}\\ b_{i,t}=\mathbb{P}(c_{i}|x_{1:t}), \end{array}$$

where *b_i_*_,_*_t_* is the belief in (or, equivalently, the posterior probability of) the *i*th context given previously observed inputs. ${\vec{B}}_{t}$ then forms the posterior distribution over all context hypotheses. The prediction equation (Eq. 1) is therefore revised to consider unknown dynamics in the input, integrating context-specific predictions (Eq. 2) weighted by their beliefs (Eq. 3), as follows:

(4) $$\begin{array}{l} \mathbb{P}(x_{t+1}|x_{1:t})=\sum_{i=1}^{M}\mathbb{P}(x_{t+1}|c_{i},x_{1:t})\mathbb{P}(c_{i}|x_{1:t})\\ \;=\sum_{i=1}^{M}\mathbb{P}(x_{t+1}|{\hat{\theta }}_{i,t})\mathbb{P}(c_{i}|x_{1:t})\\ \;=\sum_{i=1}^{M}p_{i,t}b_{i,t}, \end{array}$$

where the unknown dynamics of the input are treated in a Bayesian fashion by “integrating out” the unknown context.

Figure 2*a* illustrates the main processing stages of the model for a single time step. Upon observing the new input *x_t_*_+1_, the model first computes the set of predictive probabilities ${\vec{P}}_{t}$ using the collected statistics ${\vec{\Theta }}_{t}$ (Fig. 2*a*, Predict). The model then incrementally updates the following two quantities (Fig. 2*a*, Update): the beliefs ${\vec{B}}_{t}$ are updated with new evidence from ${\vec{P}}_{t}$ based on how well *x_t_*_+1_ was predicted under each context hypothesis; and the set of statistics ${\vec{\Theta }}_{t}$ is updated with the newly observed input *x_t_*_+1_. These are in turn used for predicting the subsequent input at time *t* + 2, and so on (Skerritt-Davis and Elhilali, 2018).

As shown in Figure 2*a*, the model emits two outputs at different processing stages, and they each reflect different levels of uncertainty and dynamics in the input.

Surprisal is a local measure of probabilistic mismatch between the model prediction and the just observed input:

(5) $$S_{t+1}=-\log\mathbb{P}(x_{t+1}|x_{1:t}),$$

where *S_t_*_+1_ is the surprisal at time *t* + 1, based on the predictive probability of *x_t_*_+1_ from Equation 4. Observations with low predictive probability have high surprisal, observations with high probability have low surprisal, and observations predicted with probability 1 (i.e., completely predictable) have zero surprisal. Relating to concepts from information theory, this measure reflects the information gained from observing *x_t_*_+1_ given its context (Samson, 1953).

Belief change is a global measure of statistical change in the input sequence. If the new input *x_t_*_+1_ is no longer well predicted using the beliefs ${\vec{B}}_{t}$ (e.g., after a change in underlying statistics), the updated beliefs ${\vec{B}}_{t+1}$ shift to reflect the change in context inferred by the model. The belief change δ*_t_* measures the distance between these two posterior distributions before and after *x_t_*_+1_ is observed:

(6) $$\delta_{t}=D_{JS}({\vec{B}}_{t}||{\vec{B}}_{t+1}),$$

where $D_{JS}(\cdot ||\cdot )$ is the Jensen–Shannon divergence. The belief change ultimately reflects dynamics in the global statistics of the observed sequence.

We derived a change detection response from the model analogous to listener behavioral responses by applying a detection threshold τ to the maximal δ*_t_*:

(7) $$\text{Model Response}=\underset{t}{\max}(\delta_{t})\geq \tau .$$

We use this response to compare the model to listeners' behavioral responses. In addition, we use the moment when this maximal belief change occurs, along with surprisal, to examine the neural response related to different dynamics in the stimuli.

#### Statistical inference along multiple dimensions.

Now, let the input sequence *x_t_* be multidimensional with two components along separate dimensions (e.g., pitch and spatial location: $x_{t}=\{x_{t}^{P},x_{t}^{S}\}$). The extension of the D-REX model to multidimensional inputs is not trivial. In this study, we use the model as a springboard to formulate hypotheses for how statistical inference operates across multiple dimensions. In these different formulations, we explored three components of the model, illustrated in Figure 2*b* (indicated in red).

##### Statistics D.

Listeners potentially collect different statistics along different dimensions. In the model, sufficient statistics are specified by the *D* parameter, the dimensionality of the Gaussian distribution, or the temporal dependence, assumed by the model. In the proposed multidimensional model, a separate *D* parameter was used for each feature (Fig. 2*b*, Predict). Each *D* parameter took one of the following two values: with *D* = 1 the model collected lower-order statistics (mean and variance); and with *D* = 2 the model additionally collected higher-order statistics (i.e., covariance between adjacent inputs).

##### Integration stage.

Building on previous neural evidence for independent predictions along different dimensions, the model generates predictions separately along each feature. We examine two possible stages for combining across dimensions after the prediction: early-stage integration (Fig. 2, top), where predictions are combined across features before updating context beliefs; and late-stage integration (Fig. 2, bottom), where the δ*_t_* is computed separately for each feature and combined before the final decision. These two alternatives represent whether the context window for estimating statistics is inferred jointly across features (early) or independently for each feature (late).

##### Integration operator f(·,·).

We test the following four different operators for how predictive information is combined across features: two linear operators, average (AVG) and weighted AVG (wAVG), where the convex weighting across features is adapted to each listener; and two nonlinear operators, minimum (MIN) and maximum (MAX). These operators are applied at the processing level specified by the integration stage.

We examined each permutation of these attributes, yielding 32 variants of the model (2 *D* × 2 *D* × 2 stages × 4 operators). Model responses differed depending on their configuration. Figure 3 shows the belief change over time across model variants for the following three example stimuli: two change stimuli (examples 1 and 2) and one control stimulus (example 3). Note that after applying a given detection threshold to belief change Eq. (7), the model variants differ in their change detection responses and, if detected, in when the detection occurs.

Model variants were used to simulate listener responses in Experiments nSP and nTP. After fitting model parameters for each variant to the behavior of each participant (described in the next section), model variants were evaluated by comparing listener responses (Fig. 2*b*, right) and model responses (Eq. 7) to the same stimuli.

#### Model fitting

For each model variant, parameters were fit to individual listeners in Experiments nSP and nTP. In addition to the decision threshold τ, there are two parameters of the model that reflect neural constraints specific to each listener: the memory parameter *M* sets an upper bound on the context window (and the number of context hypotheses), and an observation noise parameter *N* sets a lower bound on prediction uncertainty, adding independent Gaussian noise with variance *N* to the predictions. These parameters represent plausible constraints on perception known to vary across individuals, with the former representing working memory capacity (Just and Carpenter, 1992; Conway et al., 2001), and the latter, perceptual fidelity (Wightman and Kistler, 1996; Kidd et al., 2007). Models with early-stage integration have a single memory parameter, because of shared context beliefs across features; models with late-stage integration have two memory parameters (one for each feature). All models have two observation noise parameters and a single decision threshold.

A grid search with 95,000 iterations was used to find parameters *M*, *N*, and τ (memory, observation noise, and decision threshold, respectively) that best replicated listener behavior for each model variant. The model detection rate (i.e., percentage of trials wherein a change was detected, using Eq. 7) in each condition was collected for each iteration in the search procedure, and the parameters resulting in the least mean squared error (MSE) in detection rate across conditions between model and listener behavior was selected. A modified hinge loss was then used to compute goodness-of-fit for each model: this loss function penalized both incorrect model responses and correct responses close to threshold (i.e., correct with low certainty), thus rewarding models with decision signals far from threshold (i.e., correct with high certainty). Note that in this comparison, ground truth is not whether there was a change in the stimulus itself, but whether the individual listener detected a change. To determine the best model variant, *t* tests were used to compare the minimum loss between the top two performing models across subjects. Cross-validation was used to confirm the model fitting above was not susceptible to overfitting: a random partition of half of experiment trials was used to fit the model as described above, and the model was evaluated using the held-out partition. Cross-validation results were averaged over 10 iterations to reduce noise from fitting to behavior estimated over a smaller sample size. In the exploration of fitted model parameters, multiple linear regression was used to establish any linear relationship between subject detection performance and model parameters.

### Electroencephalography

#### Data recording and preprocessing

EEG data in Experiments nSP and nTP were recorded using the ActiveTwo System (BioSemi) with 64 electrodes placed on the scalp according to the international 10–20 system, along with 2 additional electrodes specified by the BioSemi system used as online reference for common-mode rejection. Data were recorded at a sampling rate of 2048 Hz.

For each subject, EEG data were preprocessed with custom scripts in MATLAB using the Fieldtrip toolbox (www.fieldtriptoolbox.org; Oostenveld et al., 2011). Bad channels were identified by eye and removed before proceeding with preprocessing. Continuous EEG was filtered to 0.3–100 Hz (two-pass fourth-order Butterworth filter for high-pass, and sixth-order Butterworth filter for low-pass) and resampled to 256 Hz. Data were then cleaned in the following three stages: the Sparse Time Artifact Removal (STAR) algorithm was used to remove channel-specific artifacts (de Cheveignè, 2016), Independent component analysis was used to remove artifacts because of eye movement and heartbeat, and missing channels were interpolated using spline interpolation. The cleaned data were then epoched by melody trial (−1 to 8 s, relative to melody onset), rereferenced to the average of all 64 scalp electrodes, and baseline corrected to the 1 s window preceding melody onset. Epochs with power exceeding 2 SDs from the mean were removed from further analysis (on average, 3.8% of trials were excluded in nSP, 5% of trials in nTP).

#### Data analysis

We further epoched neural responses to −1000 to 1500 ms around tone onsets according to model outputs (surprisal and maximal belief change) to examine neural responses time locked to predictive events determined by the model.

In an oddball-like analysis, the EEG response was averaged over nine frontocentral electrodes (Fz, F1, F2, FCz, FC1, FC2, Cz, C1, and C2) to maximize auditory-related responses. High- and low-surprisal events were defined as tones with overall surprisal above the 95th percentile and below the 5th percentile, respectively. Tone epochs within each surprisal bin were averaged, and the high-surprisal response was subtracted from the low-surprisal response to yield a difference wave.

To examine the linear relationship between the EEG response magnitude and surprisal, tone epochs across all stimuli were split into 40 bins according to overall surprisal, and tone epochs with power exceeding 2 SDs from the mean were excluded from analysis (average bin size per subject, 185 epochs). The average response across tone epochs within each bin was calculated, and the cumulative response magnitude was computed in the window 80–150 ms after tone onset and plotted against the average surprisal within each bin. A similar analysis was performed using the individual surprisal along each feature using 128 bins (average bin size per subject, 66 epochs), where the bins were determined by bifurcating the 2-D surprisal space across all tones. In both analyses, the linear relationship between surprisal and neural response was measured using linear regression.

We examined the neural response time locked to high surprisal and to maximal belief change in two time windows: 80–150 and 300–800 ms. In each window, 10 channels with the largest amplitude in the grand average (5 positive polarity, 5 negative polarity) were selected for statistical analysis. For each subject, response magnitude was measured as the decibel root mean square (rms) amplitude across channels averaged over the time window relative to a baseline window (−152 to −82 and −630 to −130 ms for the early and late windows, respectively). The significance of neural response relative to baseline was determined using *t* tests.

### Data availability

Source code is available at: https://github.com/JHU-LCAP/DREX-model.

## Results

We first present behavioral results and their consequences for multidimensional predictive processing. We then further explore the computational implications of the behavioral responses using the model. Finally, we use the model to interpret neural responses.

### Perceptual experiments

Listeners were tasked with detecting changes in the statistical properties of a sequence of complex sounds varying along the following two perceptual features: in Experiments SP and nSP, stimuli varied in spatial location (S) and pitch (P), as denoted by the naming convention; in Experiments TP and nTP, stimuli varied in timbre (T) and pitch (P). Results within each set of features are presented side by side for comparison.

#### Detection performance improves with feature conjunction

Figure 4 shows detection performance in psychophysics Experiments SP (left) and TP (right). To establish whether listeners integrated information across features to perform the change detection task, we compared single- and both-change conditions, with the nonchanging feature at low entropy (excluding mid-entropy conditions; Fig. 4, checkered bars).

In Experiment SP, an ANOVA with one within-subject factor (three conditions) showed strong significant differences between conditions (*F*_(2,28)_ = 12.07, *p* = 0.0002), with *post hoc* paired *t* tests confirming the effect between Both and each single-change condition (Both vs Pitch: *t*_(14)_ = 6.12, *p* < 0.0001; Both vs Spatial: *t*_(14)_ = 4.64, *p* = 0.0004; Fig. 4, bracket across solid and striped bars). In addition, a more stringent test showed that for each subject, performance in the Both condition was significantly better than the highest of the two single-change conditions [Both vs max(Pitch, Spatial): *t*_(14)_ = 3.70, *p* = 0.0024].

We found the same effects in Experiment TP. The ANOVA showed strong differences between change conditions (*F*_(2,28)_ = 23.74, *p* < 0.0001), with *post hoc* paired *t* tests confirming the effect between Both and each single-change condition (Both vs Pitch: *t*_(14)_ = 7.77, *p* < 0.0001; Both vs Timbre: *t*_(14)_ = 3.35, *p* = 0.0047). The more stringent test also showed that each subject performed significantly better in the Both condition compared with the maximum of the single-change conditions [Both vs max(Pitch, Timbre): *t*_(14)_ = 3.01, *p* = 0.0093].

We replicated the same analysis for behavioral responses in the EEG Experiments nSP and nTP (not shown in figure). Listeners performed the same change-detection task, with the only difference being the exclusion of the mid-entropy conditions (Fig. 1, checkered bars). We observed the same behavioral effects as above in the EEG experiments: detection performance increased in the Both condition relative to each of the single-change conditions [nSP: Both vs max(Spatial, Pitch), *t*_(17)_ = 4.86, p = 0.00,015; nTP: Both vs max(Timbre, Pitch), *t*_(17)_ = 3.29, *p* = 0.0043].

If listeners were processing each feature completely independently, we would expect performance in the Both condition to be, at most, the maximum of the two single-change conditions. Instead, the apparent increase in detection performance suggests that listeners can flexibly integrate predictive information when corroborative evidence across features is available.

#### Higher entropy in uninformative feature increases false alarms but not missed detections

In a second analysis of Experiments SP and TP, we looked at whether the uninformative (i.e., nonchanging) feature could disrupt change detection in the informative (i.e., changing) feature. We compared performance in the single-change conditions when the nonchanging feature was low entropy versus mid entropy (excluding the Both condition; Fig. 4, striped bars).

In Experiment SP, an ANOVA with two within-subject factors (two levels of changing feature × two levels of entropy of nonchanging feature) showed a significant main effect of entropy (*F*_(1,42)_ = 5.01, *p* = 0.031), and no effect of changing feature (*F*_(1,42)_ = 1.15, *p* = 0.29) or interaction (*F*_(1,42)_ = 1.12, *p* = 0.30; Fig. 4, bracket across solid and checkered bars). Interestingly, *post hoc t* tests showed that the decrease in performance was because of an increase in false alarms (FAs; Pitch/Spatial entropy: low/low vs low/mid, *t*_(14)_ = –7.44, *p* < 0.0001); low/low vs mid/low, *t*_(14)_ = –2.48, *p* = 0.013) and not a decrease in hit rates (same ANOVA as above applied to hit rates: entropy: *F*_(1,42)_ = 2.82, *p* = 0.10; feature: *F*_(1,42)_ = 0.44, *p* = 0.51; interaction: *F*_(1,42)_ = 0.55, *p* = 0.46).

We found similar effects in Experiment TP. The ANOVA showed a significant main effect of entropy (*F*_(1,42)_ = 8.00, *p* = 0.0071) and no interaction effect (*F*_(1,42)_ = 0.28, *p* = 0.60), but it did show a main effect of changing feature (*F*_(1,42)_ = 32.03, *p* < 0.0001). This difference between the Pitch and Timbre conditions likely reflects a difference in task difficulty because of stimulus design, rather than a persistent effect because of the features themselves or an interaction between the two. As for the main effect of nonchanging entropy, *post hoc t* tests again showed the decrease in detection performance was because of an increase in FAs (Pitch/Timbre entropy: low/low vs low/mid, *t*_(14)_ = –5.91, *p* < 0.0001); low/low vs mid/low, *t*_(14)_ = –3.93, *p* = 0.00,075) and not a decrease in hit rates with higher entropy (same ANOVA as above applied to hit rates: entropy: *F*_(1,42)_ = 3.5, *p* = 0.068; feature, *F*_(1,42)_ = 29.48, *p* < 0.0001; interaction, *F*_(1,42)_ = 1.75, *p* = 0.19).

The uninformative (nonchanging) feature did in fact affect overall detection performance, where higher entropy led to increased FAs; meanwhile, the detection of changes in the informative feature (i.e., hit rates) was not affected. Because stimulus conditions were randomized from trial to trial, listeners did not know *a priori* which features might change. If statistics were collected jointly across features, we would expect higher entropy in any feature to yield poorer statistical estimates, leading to higher FAs and lower hit rates. However, differences in the uninformative feature did not disrupt listeners' ability to track statistics in the informative feature. This result suggests that statistics are collected independently along each feature rather than jointly between features, and integration across features occurs after statistical estimates have been formed.

### Computational model

Behavioral results so far demonstrate that listeners collect statistics independently along multiple features and then integrate across features at some later processing stage, begging the question of how this combination occurs. To answer this, we formulated many possible models to appraise different hypotheses for the underlying computational mechanism that could lead to listener behavior. These model variants explore the following three aspects of multifeature predictive processing: (1) the statistics collected along each feature; (2) the processing stage at which integration occurs; and (3) the function or operator used to combine across features.

#### Model comparison to listener behavior

Figure 5 shows the loss by model (rows) and subject (columns) after the fitting procedure for Experiments nSP and nTP. For each experiment, models are ordered by decreasing average loss (Fig. 5, top row, minimum average loss), and subjects are ordered by increasing detection performance *d^′^* (Fig. 5, right column, highest *d^′^*). Model variants are labeled according to the configuration illustrated in Figure 2*b*: stage_DXX_operator, where XX specifies the statistics (1 or 2) used for each feature. For example, in Experiment nSP in Figure 5, the Early_D12_MAX model uses early-stage integration, *D* = 1 for pitch, *D* = 2 for spatial, and the MAX operator for integration.

The column to the right of each fit matrix in Figure 5 shows the average loss across all subjects. The model labels reveal high agreement in the top-performing models fit across Experiment nSP and Experiment nTP—in fact, the ordering of the top 11 models is identical across experiments. Notably, model Late_D22_MAX yields the best fit on average across all subjects for both experiments. Specifically, Late_D22_MAX has a significantly lower loss (i.e., better fit) across subjects when compared with the next best model, Early_D22_MIN, in both experiments (nSP: *t*_(17)_ = –3.82, *p* = 0.0014; nTP: *t*_(17)_ = –3.63, *p* = 0.0021).

With the poorer fitting models in the lower half of Figure 5, model variants with Early-MAX or Late-MIN have a fit loss near chance. This is not surprising given that both are less sensitive to changes: the Early-MAX models only detect changes when both features violate prediction, and similarly the Late-MIN models require the change signal of both features to cross threshold. Neither of these types of models fit listener behavior well. Additionally, models with lower-order statistics (i.e., *D* = 1) in one or both features tend to have poorer fits (and higher loss).

To examine the robustness of the model fitting, we performed cross-validation where the model was fit to a random partition of half of experiment trials, and this fitted model was then compared with listener behavior using task performance (*d^′^*) on the held-out trials. Figure 6 shows model performance on the test partition for four model variants plotted against listener behavior. The four models—Late_D22_MAX, Late_D22_MIN, Late_D11_MAX, and Early_D11_MAX—were selected to demonstrate how tweaks in the model configuration lead to drastically different replication results. For each model variant, a linear regression with explained variance (*R*^2^) is overlaid onto the data, and the diagonal line indicates an ideal model (i.e., model performance matching listener behavior). The best-fitting model variant from the comparison above (Late_D22_MAX) explains a high amount of variability in listener behavior across performance levels and has low MSE, approaching the diagonal in both experiments (SP: *R*^2^ = 0.96, MSE = 0.14; TP: *R*^2^ = 0.98, MSE = 0.24). By comparison, the performance of higher-perforning subjects cannot be matched by the Late_D22_MIN model (SP: *R*^2^ = 0.20, MSE = 1.02; TP: *R*^2^ = 0.34, MSE = 2.11), or by the Late_D11_MAX model (SP: *R*^2^ = 0.45, MSE = 1.04; TP: *R*^2^ = 0.40, MSE = 2.30). And the Early_D11_MAX model is not able to perform the task at all, with performance below chance (SP: *R*^2^ = 0.21, MSE = 3.59; TP: *R*^2^ = 0.18, MSE = 7.40). This cross-validation analysis confirms the results above showing that the Late_D22_MAX model is able to closely replicate listener behavior in both Experiments SP and TP.

Together, these results suggest that for both spectral and spatial features, listeners track higher-order statistics separately along each feature and integrate at a later stage, making a nonlinear change decision based on the feature with the most evidence for change. In later analyses, we use this fitted model to guide analysis of neural responses.

#### Model interpretation of individual differences

Looking closer at variability in model loss across individuals in Figure 5, some patterns emerge across Experiments nSP and nTP. For better performing subjects (Fig. 5: *d^′^* > 1, right side of each image), there is high agreement in loss across all model variants. For poorer performing subjects (Fig. 5, left side of each image), there is more variability in model fit across subjects, with some model variants with higher overall loss fitting individual subjects quite well. For example, in Experiment nSP (Fig. 5, left), the Late_D12_MAX model has loss near chance for subjects with *d^′^* > 1, but for subjects with *d^′^* < 1, loss is near zero. Interpreting through the lens of the model, this suggests individualized listening strategies, possibly reflecting differences in the inherent ability to track statistics in sound sequences. Unfortunately, in this work we are unable to disentangle the effects because of the listening strategy from noise effects because of lack of attention, deficient task understanding, or fatigue; however, there are avenues for using the model to investigate this in future work.

We can also examine how individual differences are explained by the model parameters fit to each subject. Using the Late_D22_MAX model, the “best” overall model, we tested for correspondence among the four perceptual parameters (memory and observation noise for each feature) and detection performance across listeners. In Experiment nSP, a multiple linear regression explained 82% of the variance in *d^′^* and showed strongly significant correlation between both memory parameters and detection performance (*M_S_*, *p* = 0.0070; *M_P_*, *p* = 0.0004) and no significant correlation between the observation noise parameter and performance in either feature (*N_S_*, *p* = 0.82; *N_P_*, *p* = 0.33), where *M_S_* and *M_P_* are the memory parameter for spatial location and pitch, respectively, and *N_S_* and *N_P_* are the accompanying observation noise parameter for each feature.. We see similar results in Experiment nTP, with the perceptual parameters accounting for 81% of the variance in *d^′^* and significant correlation between the spatial memory parameter with weaker significance in the pitch memory parameter (*M_T_*, *p* = 0.0009; *M_P_*, *p* = 0.0975; *N_T_*, *p* = 0.87; *N_P_*, *p* = 0.54), where *M_T_* and *N_T_* are the memory and observation noise parameters for timbre, respectively. Figure 7 shows the fitted memory parameters for each feature plotted against overall *d^′^* for Experiments nSP (left) and nTP (right), along with the multiple linear regression. This result suggests that the differences in behavior across listeners in Experiments nSP and nTP could be because of differences in memory capacity rather than difference in perceptual fidelity (as represented by observation noise), where better performing subjects use higher memory capacity for statistical estimation in each feature. We stress, however, that the model parameters are indirect measures of memory capacity and perceptual fidelity, but these questions could be further probed in future work.

We additionally tested for correlations between memory parameters across feature. Linear regression showed significant correlations in memory across features in both experiments (nSP: ρ = 0.53, *p* = 0.0232; nTP: ρ = 0.61, *p* = 0.0076). This result holds implications for the independence of neural resources used in statistical predictive processing: While predictions occur separately across features, this suggests that the quality of statistical estimates (as embodied by the memory parameter of the model) is linked across features.

### EEG brain responses

The model simulates predictive processing moment by moment, giving a window into the underlying processes that cannot be observed through behavior. In this section, we use the Late_D22_MAX model to guide analysis of neural responses in Experiments nSP and nTP.

Two model outputs were used to specify epochs for trial averaging: surprisal, the local measure of deviance between each observation and its prediction; and maximal belief change, the global measure of melody-level statistics when the largest change in beliefs occurs in each trial. Note that there are distinct surprisal responses for each feature (e.g., each tone in the melody elicits a surprisal in pitch and a surprisal in spatial location from the model). In comparison, the maximal belief change occurs once in each trial and reflects more global statistical processing of the stimulus sequence.

#### Neural response magnitude increases with local surprisal

We used model surprisal to perform an oddball-like analysis of neural responses. While this type of analysis typically relies on deterministic patterns to define “deviant” and “standard” events, without such structure we use surprisal from the model to guide identification of tones that fit predictions well and those that do not. First, we use an overall measure of surprisal to define deviant and standard by summing surprisal across features (e.g., $S_{t}=S_{t}^{P}+S_{t}^{S}$, where $S_{t}^{P}$ and $S_{t}^{S}$ are the tone-by-tone surprisal from pitch and spatial location, respectively; Eq. 5). We compared the neural response time locked to high-surprisal tones to the response time locked to low-surprisal tones, where high and low were defined as the top and bottom 5%, respectively, for each subject. In this analysis, we averaged the EEG response across frontocentral electrodes typically used in auditory analyses (according to 10–20 system: Cz, C1, C2, FCz, FC1, FC1, Fz, F1, and F2).

Figure 8*a* shows the grand average response to high- and low-surprisal tones along with their difference wave for Experiments nSP and nTP. High-surprisal tones elicit a larger magnitude response relative to low-surprisal tones, as can be seen in deviations in the difference wave from 0 µV at typical N1 and P2 time windows. Topography in Figure 8*a* shows the amplitude of differential response in the 80–150 ms window after tone onset, along with channels used in this analysis. Note the oscillations in the grand average response are entrained to tone onsets (every 116 ms); the response to high-surprisal tones augments this obligatory onset response.

To determine whether there is a linear relationship between the overall surprisal (*S_t_*) and the neural response, we took advantage of surprisal as a continuous measure of probabilistic deviance to bin tones across all trials into 40 equal-sized bins by overall surprisal. We then averaged the neural response within each bin across subjects and across tone epochs and extracted the neural response magnitude 80–150 ms after tone onset (corresponding to the typical N1/mismatch negativity (MMN) time window; Fig. 8*a*, overlay on difference wave). Figure 8*b* shows EEG magnitude plotted against surprisal in each bin. Linear regression showed an increase in EEG magnitude with increasing surprisal in both experiments (nSP: *R*^2^ = 0.62, *p* < 0.0001; nTP: *R*^2^ = 0.54, *p* < 0.0001). However, nonparametric tests using Spearman correlations partially contradicted the significance of these results for Experiment nTP (*p* = 0.14), while the effect in Experiment nSP remained highly significant (*p* = 0.0006).

We further examined this linear relationship in an extended analysis using the feature-specific surprisal (e.g., $S_{t}^{P}$ and $S_{t}^{S}$). For each subject, tone epochs were binned into 128 equal-sized bins in the 2-D space spanned by surprisal along each feature, and the neural response was averaged within each bin over epochs and subjects. Figure 8*c* displays EEG magnitude for each bin at the average surprisal along each feature. Multiple linear regression shows a strongly significant correlation between EEG magnitude and surprisal in both experiments (nSP: *R*^2^ = 0.41, *p* < 0.0001; nTP: *R*^2^ = 0.38, *p* < 0.0001) with EEG magnitude significantly increasing with surprisal along both features (nSP: pitch surprisal, *p* = 0.0124; spatial surprisal, *p* < 0.0001; nTP: pitch surprisal, *p* = 0.0272; timbre surprisal, *p* < 0.0001).

Going beyond previous work showing linear superposition of deviance responses in oddball paradigms (Takegata et al., 1999), these results show that the neural response magnitude increases proportionally with the level of surprisal along each feature, which then combines linearly in the EEG response recorded at the scalp. This effect cannot be measured from stimulus properties alone nor by behavior, requiring a model to estimate the local surprisal of each tone along each feature given its context.

#### Distinct responses to local surprisal and global statistical change

We next examined neural responses aligned to high-surprisal events alongside responses aligned to the maximal belief change, where the former represents local prediction mismatch and the latter represents global statistical change in the stimulus. High surprisal is again defined as tones with overall surprisal (e.g., $S_{t}=S_{t}^{P}+S_{t}^{S}$) in the top 5%. Maximal belief change is the moment when the δ*_t_* reaches its maximum across the melody trial (Eq. 6). Note that by aligning to model responses before trial averaging, the temporal position of the motor response relative to time = 0 is shuffled, thereby reducing confounds because of motor preparation.

**Figure 9.**
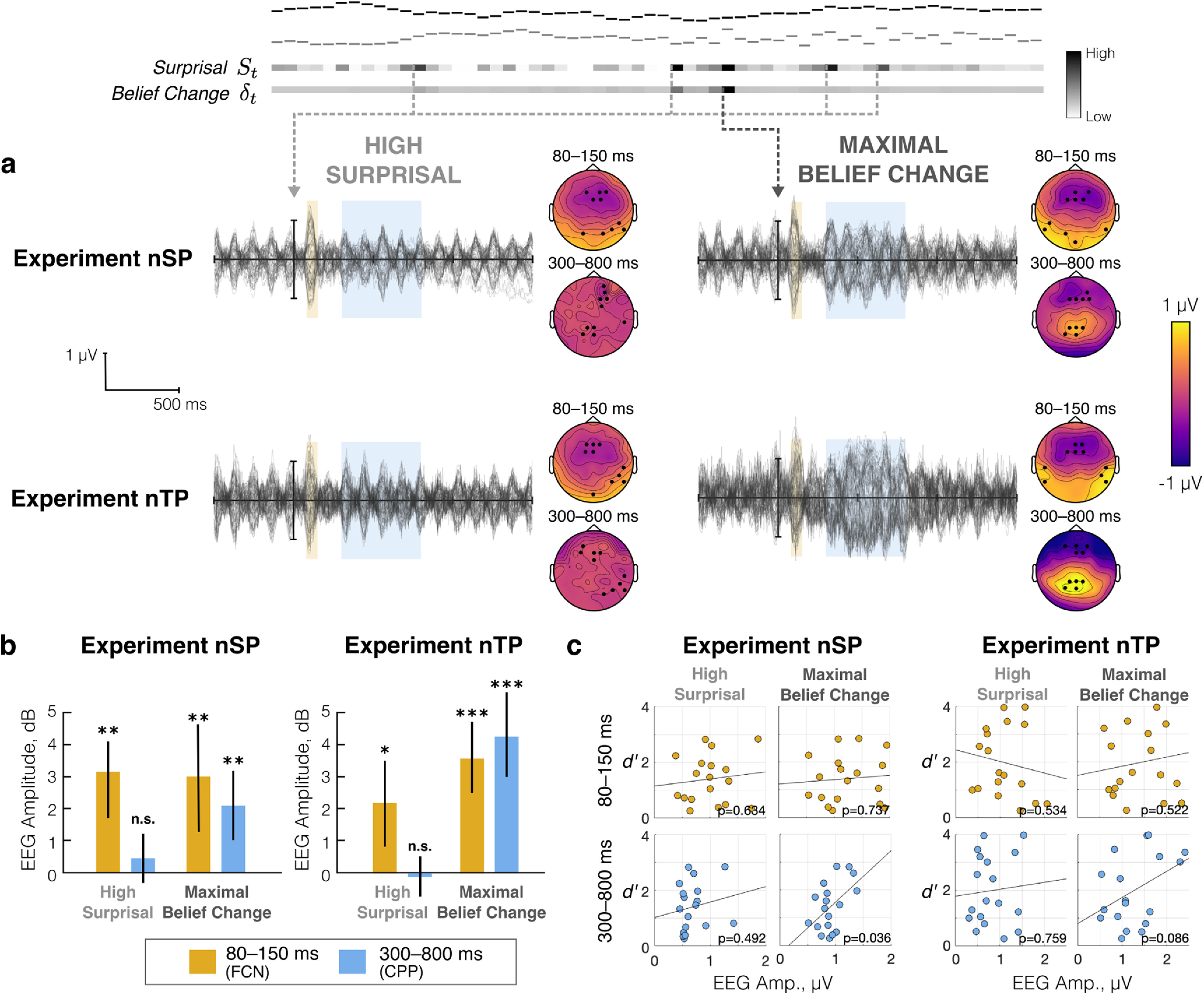
Neural response aligned to model outputs. Illustration of example stimulus with model outputs above: moments of high surprisal and maximal surprisal (black, high) used to align epochs for time averaging. ***a***, Grand-average responses for Experiments nSP (top) and nTP (bottom). Shaded regions indicate two time windows of interest, with topography to the right showing the average response amplitude within each time window at each channel relative to baseline. Highlighted channels used in statistical analysis. ***b***, rms amplitude in decibels relative to baseline in each time window (color) at each aligning event (horizontal axis). Error bars indicate 95% bootstrap confidence interval across subjects. ***c***, Response amplitude in each time window at each aligning event plotted against detection performance (*d^′^*) across subjects.

Figure 9*a* (top) shows an illustration of this analysis with an example stimulus and its model outputs, *S_t_* and δ*_t_*. Dotted lines show moments used to align epochs for each type of event. High-surprisal events can occur at multiple points within the same melody stimulus, while there is only one maximal belief change. Note that when an epoch qualified as both a high-surprisal and maximal belief change, it was excluded from the high-surprisal events to keep the epochs in each average response distinct. For each subject, the neural response was averaged for each aligning event (i.e., high-surprisal and maximal belief changes) across epochs from all melody trials.

Below the illustration, Figure 9*a* shows the grand average neural response across subjects for all 64 channels time locked to the two aligning events, high surprisal (left) and maximal belief change (right), in Experiments nSP (top) and nTP (bottom). Topography to the right of each grand average show two responses that emerge in the highlighted time windows after alignment: an early frontocentral negativity (FCN) with a latency of 80–150 ms (the same surprisal response examined above), and a later (and much slower) centroparietal positivity (CPP) with a latency of 300–800 ms.

To determine whether the neural response is significantly larger in these two time windows, we compared the cumulative rms amplitude of the neural response to baseline amplitudes in windows at the same cyclic position relative to neural entrainment (−152 to −82 and −630 to −130 ms for the early and late windows, respectively). In each time window, 10 channels with the largest magnitude in the grand average (5 with positive polarity, 5 with negative polarity) were selected for within-subjects analysis; selected channels for each response are highlighted in the topography in Figure 9*a*. Figure 9*b* shows decibel amplitude in Experiments nSP (left) and nTP (right). In both experiments, the neural response amplitude increased significantly in the early window after high-surprisal tones (nSP: *t*_(17)_ = 3.88, *p* = 0.0012; nTP: *t*_(17)_ = 2.45, *p* = 0.0253) and after the maximal belief change (nSP: *t*_(17)_ = 2.93, *p* = 0.0093; nTP: *t*_(17)_ = 4.86, *p* = 0.0001). Note that maximal belief change often coincides with high surprisal (Fig. 9, top), so this result is not altogether “surprising.” However, in the later window, the neural response only significantly increased after maximal belief change (nSP: *t*_(17)_ = 3.02, *p* = 0.0076; nTP: *t*_(17)_ = 4.98, *p* = 0.0001), with no significant increase in amplitude after other high-surprisal moments in both experiments (nSP: *t*_(17)_ = 1.05, *p* = 0.31; nTP: *t*_(17)_ = –0.43, *p* = 0.67).

Finally, we examined the relationship between these effects and behavioral performance in the change detection task in Experiments nSP and nTP. Figure 9*c* shows the overall *d^′^* value for each subject (vertical axis) plotted against the neural response amplitude (horizontal axis) in each time window (by row) at each aligning event (by column). Linear regression analysis showed no significant correlation between neural responses and behavior in the early time window at either aligning event. At the maximal belief change, however, correlations between the neural response amplitude in the late time window (i.e., the CPP response) and behavior is significant in Experiment nSP (*R*^2^ = 0.2, *p* = 0.036) and is marginally significant in Experiment nTP (*R*^2^ = 0.12, *p* = 0.086).

Together, these results suggest distinct underlying neural computations leading to the FCN and CPP effects. The FCN effect is elicited by any high-surprisal event. Moments of maximal belief change are a subset of these events, where incoming observations no longer fit with current statistical estimates, resulting in poor predictions and higher surprisal. The surprisal response, as shown in the previous analysis, is elicited independently along each feature and combines linearly for multidimensional sounds. The CPP effect, on the other hand, occurs only at the maximal belief change, suggesting that this response relates to global contextual processing after integrating nonlinearly across features. Additionally, this CPP effect is weaker for poorer performing subjects (Fig. 9*c*), possibly reflecting individual differences in integration strategies or memory capacity for statistical estimation.

## Discussion

Sound sources in natural environments vary along multiple acoustic dimensions, yet how the brain integrates these features into a coherent auditory object is an open question. Our approach combined psychophysics, computational modeling, and EEG to probe the mechanisms behind feature integration in predictive processing. Importantly, we used a stochastic change detection paradigm to approximate the challenges and uncertainty encountered in natural environments, where regularities emerge at unknown times and along unknown perceptual dimensions.

Through behavioral results, we demonstrated that listeners have access to a joint representation to perform the stochastic change detection task, flexibly combining evidence for statistical change across multiple features. To illuminate how this joint representation is constructed, we used a computational model grounded in Bayesian accounts of statistical predictive coding in the brain (Knill and Pouget, 2004; Tenenbaum et al., 2006; Daunizeau et al., 2010; Pieszek et al., 2013; Wilson et al., 2013). This model embodies several theoretical principles of predictive processing: that the brain maps sensory inputs onto compact summary statistics (Brady et al., 2009; McDermott et al., 2013); that the brain entertains multiple hypotheses or interpretations of sensory information (Mumford, 1991); and that the brain incrementally updates its predictions over time based on evidence from new inputs (Darriba and Waszak, 2018). The D-REX model and its multifeature extension presented above represent a computational instantiation of these theoretical principles that can be used to interpret experimental results.

We formulated multiple possible implementations for statistical prediction and integration. Using experimental data to fit these model variants to each subject, our analysis suggests that listeners independently collect higher-order statistics and infer context along multiple dimensions, integrating across dimensions at a later stage. We additionally used this best model to interpret variability in behavior across listeners, where detection performance ranged from near chance to near ceiling. A high degree of variability in listener behavior could be explained by the memory parameter of the model, which represents working memory capacity used to estimate statistics along each feature known to vary from person to person (Just and Carpenter, 1992; Conway et al., 2001; Kidd et al., 2007). Interestingly, the fitted memory parameters correlated across features, suggesting that listeners are estimating statistics under the same neural resource constraints across dimensions. Alternatively, variability in behavior could be because of different listening strategies or resolution of statistical representation (*D* = 1 or 2), particularly for lower-performing subjects. Worth noting is that the same lower-performing subjects (*d^′^* < 1) also reveal weaker centroparietal late activity in response to maximal belief change of the melody, which may underlie limited predictive tracking or sluggish cross-feature integration of statistical beliefs. The lack of any correlation between surprisal brain responses and perceptual performance (Fig. 9*c*) argues against poor performance being because of weaker deviance tracking at the level of individual features. Naturally, the source of these individual differences needs further investigation in future work with better tailored experimental paradigms.

That being said, it is clear from the neural responses that the brain multiplexes two types of responses that can be defined in terms of predictive processing. The FCN is an MMN-like response, having similar characteristics to the response to deviants in oddball experiments (Takegata et al., 2001; Pakarinen et al., 2007; Garrido et al., 2013). Borrowing terminology from the oddball paradigm, in our analysis we used the model to define deviant events in our stochastic stimuli. These high-surprisal events were followed by the FCN response (changepoint or not), signifying a local, tone-level response because of mismatch between the immediate sensory input and internal predictions. Furthermore, we found that the response magnitude was proportional with surprisal in each feature independently, agreeing with similar results in the literature using less stochastic stimuli (Paavilainen et al., 2001; Vuust et al., 2016). This parallel tracking likely leverages the topographic organization in auditory cortex along different features (Schreiner, 1992; Read et al., 2002).

The CPP, on the other hand, is later, having similar latency and topography to the P3b response, which has been linked to context updating in working memory because of expectation violations (Donchin and Coles, 1988; Polich, 2007; Romero-Rivas et al., 2018; Darriba and Waszak, 2018). Additionally, in contrast to the MMN response, the P3b is associated with changes in global regularities encompassing higher-order statistics (Bekinschtein et al., 2009; Wacongne et al., 2011; Chennu et al., 2013) and more complex stimuli (Chernyshev et al., 2016). Our interpretation agrees with these previous results: the CPP effect follows maximal changes in the context beliefs, the equivalent of context updating within the terminology of our model, and these shifts reflect broader changes in the statistics of the melody after integrating across features, rather than a response to a single tone or a single feature.

Finally, all of our results, from behavior to modeling to EEG, were consistent across two sets of experiments, each using a different combination of features. Where in one set of experiments (SP and nSP) the features were spectral and spatial, the second set (TP and nTP) used features that were both spectral in nature, countering the argument that these results were because of distinct what/where pathways in the brain (Murray and Spierer, 2009). Instead, these results support a domain-general statistical predictive coding machinery in the brain that operates in parallel along multiple perceptual features to tackle the uncertainty present in complex environments.

## References

[B1] Agus TR, Thorpe SJ, Pressnitzer D (2010) Rapid formation of robust auditory memories: insights from noise. Neuron 66:610–618. 10.1016/j.neuron.2010.04.014 20510864

[B2] Algazi VR, Duda RO, Thompson DM, Avendano C (2001) The CIPIC HRTF database. In: Proceedings of the 2001 IEEE workshop on applications of signal processing to audio and acoustics, pp 99–102. New Paltz, NY: IEEE.

[B3] Allen EJ, Burton PC, Olman CA, Oxenham AJ (2017) Representations of pitch and timbre variation in human auditory cortex. J Neurosci 37:1284–1293. 10.1523/JNEUROSCI.2336-16.2016 28025255 PMC5296797

[B4] Angelaki DE, Gu Y, DeAngelis GC (2009) Multisensory integration: psychophysics, neurophysiology, and computation Curr Opin Neurobiol 19:452–458. 19616425 10.1016/j.conb.2009.06.008 PMC2749464

[B5] Attias H, Schreiner CE (1997) Temporal low-order statistics of natural sounds. Paper presented at NIPS 1996 10th Annual Conference on Neural Information Processing Systems, Denver, CO, December.

[B6] Bekinschtein TA, Dehaene S, Rohaut B, Tadel F, Cohen L, Naccache L (2009) Neural signature of the conscious processing of auditory regularities. Proc Natl Acad Sci U S A 106:1672–1677. 10.1073/pnas.0809667106 19164526 PMC2635770

[B7] Brady TF, Konkle T, Alvarez GA (2009) Compression in visual working memory: using statistical regularities to form more efficient memory representations. J Exp Psychol Gen 138:487–502. 10.1037/a0016797 19883132

[B8] Caclin A, Brattico E, Tervaniemi M, Näätänen R, Morlet D, Giard M-H, McAdams S (2006) Separate neural processing of timbre dimensions in auditory sensory memory. J Cogn Neurosci 18:1959–1972. 10.1162/jocn.2006.18.12.1959 17129184

[B9] Chennu S, Noreika V, Gueorguiev D, Blenkmann A, Kochen S, Ibáñez A, Owen A, Bekinschtein TA (2013) Expectation and attention in hierarchical auditory prediction. J Neurosci 33:11194–11205. 10.1523/JNEUROSCI.0114-13.2013 23825422 PMC3718380

[B10] Chernyshev BV, Bryzgalov DV, Lazarev IE, Chernysheva EG (2016) Distributed feature binding in the auditory modality. Neuroreport 27:837–842. 10.1097/WNR.0000000000000623 27306594

[B11] Conway ARA, Cowan N, Bunting MF (2001) The cocktail party phenomenon revisited: the importance of working memory capacity. Psychon Bull Rev 8:331–335. 10.3758/bf03196169 11495122

[B12] Conway CM, Christiansen MH (2005) Modality-constrained statistical learning of tactile, visual, and auditory sequences. J Exp Psychol Learn Mem Cogn 31:24–38. 10.1037/0278-7393.31.1.24 15641902

[B13] Creel SC, Newport EL, Aslin RN (2004) Distant melodies: statistical learning of nonadjacent dependencies in tone sequences. J Exp Psychol Learn Mem Cogn 30:1119–1130. 15355140 10.1037/0278-7393.30.5.1119

[B14] Daikhin L, Ahissar M (2012) Responses to deviants are modulated by subthreshold variability of the standard. Psychophysiology 49:31–42. 10.1111/j.1469-8986.2011.01274.x 21899557 PMC3240736

[B15] Darriba A, Waszak F (2018) Predictions through evidence accumulation over time. Sci Rep 8:494. 10.1038/s41598-017-18802-z 29323172 PMC5765034

[B16] Daunizeau J, den Ouden HEM, Pessiglione M, Kiebel SJ, Friston KJ, Stephan KE (2010) Observing the observer (II): deciding when to decide. PLoS One 5 5:e15555. 10.1371/journal.pone.0015555 21179484 PMC3001882

[B17] Degel J (2001) Implicit learning and implicit memory for odors: the inuence of odor identification and retention time. Chem Senses 26:267–280. 10.1093/chemse/26.3.267 11287387

[B18] de Cheveignè A (2016) Sparse time artifact removal. J Neurosci Methods 262:14–20. 26778608 10.1016/j.jneumeth.2016.01.005

[B19] Donchin E, Coles MG (1988) Is the P300 component a manifestation of context updating? Behav Brain Sci 11:357–374. 10.1017/S0140525X00058027

[B20] Du Y, He Y, Ross B, Bardouille T, Wu X, Li L, Alain C (2011) Human auditory cortex activity shows additive effects of spectral and spatial cues during speech segregation. Cereb Cortex 21:698–707. 10.1093/cercor/bhq136 20685854

[B21] Dyson BJ, Ishfaq F (2008) Auditory memory can be object based. Psychon Bull Rev 15:409–412. 10.3758/pbr.15.2.409 18488660

[B22] Ernst MO, Harrar V, Parise CV, Spence C (2013) Cross-correlation between auditory and visual signals promotes multisensory integration. Multisens Res 26:307–316. 10.1163/22134808-00002417 23964482

[B23] Fetsch CR, Deangelis GC, Angelaki DE (2010) Visual-vestibular cue integration for heading perception: applications of optimal cue integration theory. Eur J Neurosci 31:1721–1729. 20584175 10.1111/j.1460-9568.2010.07207.x PMC3108057

[B24] Fiser J, Aslin RN (2002) Statistical learning of higher-order temporal structure from visual shape sequences. J Exp Psychol Learn Mem Cogn 28:458–467. 12018498 10.1037//0278-7393.28.3.458

[B25] Friston KJ (2005) A theory of cortical responses. Philos Trans R Soc Lond B Biol Sci 360:815–836. 10.1098/rstb.2005.1622 15937014 PMC1569488

[B26] Frost R, Armstrong BC, Siegelman N, Christiansen MH (2015) Domain generality versus modality specificity: the paradox of statistical learning. Trends Cogn Sci 19:117–125. 25631249 10.1016/j.tics.2014.12.010 PMC4348214

[B27] Garcia-Lazaro JA, Ahmed B, Schnupp JWH (2006) Tuning to natural stimulus dynamics in primary auditory cortex. Curr Biol 16:264–271. 10.1016/j.cub.2005.12.013 16461279

[B28] Garrido MI, Sahani M, Dolan RJ (2013) Outlier responses reect sensitivity to statistical structure in the human brain. PLoS Comput Biol 9:e1002999. 10.1371/journal.pcbi.1002999 23555230 PMC3610625

[B29] Geffen MN, Gervain J, Werker JF, Magnasco MO (2011) Auditory perception of self-similarity in water sounds. Front Integr Neurosci 5:15. 10.3389/fnint.2011.00015 21617734 PMC3095814

[B30] Heilbron M, Chait M (2018) Great expectations: is there evidence for predictive coding in auditory cortex? Neuroscience 389:54–73. 10.1016/j.neuroscience.2017.07.061 28782642

[B31] Just MA, Carpenter PA (1992) A capacity theory of comprehension: individual differences in working memory. Psychol Rev 99:122–149. 10.1037/0033-295x.99.1.122 1546114

[B32] Kidd GR, Watson CS, Gygi B (2007) Individual differences in auditory abilities. J Acoust Soc Am 122:418–435. 10.1121/1.2743154 17614500

[B33] Knill DC, Pouget A (2004) The Bayesian brain: the role of uncertainty in neural coding and computation. Trends Neurosci 27:712–719. 10.1016/j.tins.2004.10.007 15541511

[B34] Krishnan S, Carey D, Dick F, Pearce M (2019) Effects of statistical learning in passive and active contexts on reproduction and recognition of auditory sequences. PsyArXiv. Advance online publication. Retrieved June 29, 2021. doi: 10.31234/osf.io/vbem8. 34582231

[B35] Levitin DJ, Chordia P, Menon V (2012) Musical rhythm spectra from Bach to Joplin obey a 1/f power law. Proc Natl Acad Sci U S A 109:3716–3720. 10.1073/pnas.1113828109 22355125 PMC3309746

[B36] Maniscalco B, Abry P, Holroyd T, Lin A, Lee JL, He BJ (2018) Neural integration of stimulus history underlies prediction for naturalistically evolving sequences. J Neurosci 38:1541–1557. 10.1523/JNEUROSCI.1779-17.2017 29311143 PMC5815353

[B37] McDermott JH, Schemitsch M, Simoncelli EP (2013) Summary statistics in auditory perception. Nat Neurosci 16:493–498. 10.1038/nn.3347 23434915 PMC4143328

[B38] Melara RD, Marks LE (1990) Interaction among auditory dimensions: timbre, pitch, and loudness. Percept Psychophys 48:169–178. 10.3758/bf03207084 2385491

[B39] Mumford D (1991) On the computational architecture of the neocortex—I. The role of the thalamo-cortical loop. Biol Cybern 65:135–145. 10.1007/BF00202389 1912004

[B40] Murphy KP (2007) Conjugate bayesian analysis of the gaussian distribution. Technical Report. University of British Columbia.

[B41] Murray MM, Spierer L (2009) Auditory spatio-temporal brain dynamics and their consequences for multisensory interactions in humans. Hear Res 258:121–133. 10.1016/j.heares.2009.04.022 19454307

[B42] Nelken I, Rotman Y, Yosef OB (1999) Responses of auditory-cortex neurons to structural features of natural sounds. Nature 397:154–157. 10.1038/16456 9923676

[B43] Oostenveld R, Fries P, Maris E, Schoffelen JM (2011) FieldTrip: open source software for advanced analysis of MEG, EEG, and invasive electrophysiological data. Comput Intell Neurosci 2011:156869. 10.1155/2011/156869 21253357 PMC3021840

[B44] Overath T, Cusack R, Kumar S, von KK, Warren JD, Grube M, Carlyon RP, Griffiths TD (2007) An information theoretic characterisation of auditory encoding. PLoS Biol 5:e288. 10.1371/journal.pbio.0050288 17958472 PMC2039771

[B45] Paavilainen P, Valppu S, Näätänen R (2001) The additivity of the auditory feature analysis in the human brain as indexed by the mismatch negativity: 1+1 approximately 2 but 1+1+1. Neurosci Lett 301:179–182. 10.1016/S0304-3940(01)01635-4 11257427

[B46] Pakarinen S, Takegata R, Rinne T, Huotilainen M, Näätänen R (2007) Measurement of extensive auditory discrimination profiles using the mismatch negativity (MMN) of the auditory event-related potential (ERP). Clin Neurophysiol 118:177–185. 10.1016/j.clinph.2006.09.001 17070103

[B47] Parise CV, Spence C, Ernst MO (2012) When correlation implies causation in multisensory integration. Curr Biol 22:46–49. 10.1016/j.cub.2011.11.039 22177899

[B48] Pearce MT, Ruiz MH, Kapasi S, Wiggins GA, Bhattacharya J (2010) Unsupervised statistical learning underpins computational, behavioural, and neural manifestations of musical expectation. Neuroimage 50:302–313. 10.1016/j.neuroimage.2009.12.019 20005297

[B49] Pickover CA, Khorasani A (1986) Fractal characterization of speech waveform graphs. Comput Graph 10:51–61. 10.1016/0097-8493(86)90068-3

[B50] Pieszek M, Widmann A, Gruber T, Schröger E (2013) The human brain maintains contradictory and redundant auditory sensory predictions. PLoS One 8:e53634. 10.1371/journal.pone.0053634 23308266 PMC3538730

[B51] Polich J (2007) Updating P300: an integrative theory of P3a and P3b. Clin Neurophysiol 118:2128–2148. 17573239 10.1016/j.clinph.2007.04.019 PMC2715154

[B52] Read HL, Winer JA, Schreiner CE (2002) Functional architecture of auditory cortex. Curr Opin Neurobiol 12:433–440. 12139992 10.1016/s0959-4388(02)00342-2

[B53] Romero-Rivas C, Vera-Constán F, Rodríguez-Cuadrado S, Puigcerver L, Fernández-Prieto I, Navarra J (2018) Seeing music: the perception of melodic 'ups and downs' modulates the spatial processing of visual stimuli. Neuropsychologia 117:67–74. 10.1016/j.neuropsychologia.2018.05.009 29753020

[B54] Samson E (1953) Fundamental natural concepts of information theory. Etc. 10:283–297.

[B55] Schmuckler MA, Gilden DL (1993) Auditory perception of fractal contours. J Exp Psychol Hum Percept Perform 19:641–660. 10.1037/0096-1523.19.3.641 8331318

[B56] Schreiner CE (1992) Functional organization of the auditory cortex: maps and mechanisms. Curr Opin Neurobiol 2:516–521. 10.1016/0959-4388(92)90190-V 1525552

[B57] Schröger E, Wolff C (1996) Mismatch response of the human brain to changes in sound location. Neuroreport 7:3005–3008. 10.1097/00001756-199611250-00041 9116228

[B58] Seriès P, Seitz AR (2013) Learning what to expect (in visual perception). Front Hum Neurosci 7:668. 24187536 10.3389/fnhum.2013.00668 PMC3807544

[B59] Shinn-Cunningham BG, Best V, Ozmeral EJ, Kopco N, Kopčo N, Shinn-Cunningham BG (2008) Object continuity enhances selective auditory attention. Proc Natl Acad Sci U S A 105:13174–13178. 10.1073/pnas.0803718105 18719099 PMC2529120

[B60] Skerritt-Davis B, Elhilali M (2018) Detecting change in stochastic sound sequences. PLoS Comput Biol 14:e1006162. 10.1371/journal.pcbi.1006162 29813049 PMC5993325

[B61] Skerritt-Davis B, Elhilali M (2019) A model for statistical regularity extraction from dynamic sounds. Acta Acust United Acust 105:1–4. 10.3813/AAA.919279 31929768 PMC6953992

[B62] Takegata R, Paavilainen P, Näätänen R, Winkler I (1999) Independent processing of changes in auditory single features and feature conjunctions in humans as indexed by the mismatch negativity. Neurosci Lett 266:109–112. 10.1016/S0304-3940(99)00267-0 10353339

[B63] Takegata R, Huotilainen M, Rinne T, Näätänen R, Winkler I (2001) Changes in acoustic features and their conjunctions are processed by separate neuronal populations. Neuroreport 12:525–529. 10.1097/00001756-200103050-00019 11234757

[B64] Takegata R, Brattico E, Tervaniemi M, Varyagina O, Näätänen R, Winkler I (2005) Preattentive representation of feature conjunctions for concurrent spatially distributed auditory objects. Brain Res Cogn Brain Res 25:169–179. 10.1016/j.cogbrainres.2005.05.006 15953710

[B65] Tenenbaum JB, Griffiths TL, Kemp C (2006) Theory-based Bayesian models of inductive learning and reasoning. Trends Cogn Sci 10:309–318. 10.1016/j.tics.2006.05.009 16797219

[B66] Thompson WF, Sinclair D (1993) Pitch pattern, durational pattern, and timbre: a study of the perceptual integration of auditory qualities. Psychomusicology 12:3–21. 10.1037/h0094121

[B67] Treisman AM, Gelade G (1980) A feature-integration theory of attention. Cogn Psychol 12:97–136. 10.1016/0010-0285(80)90005-5 7351125

[B68] Vuust P, Liikala L, Näätänen R, Brattico P, Brattico E (2016) Comprehensive auditory discrimination profiles recorded with a fast parametric musical multi-feature mismatch negativity paradigm. Clin Neurophysiol 127:2065–2077. 10.1016/j.clinph.2015.11.009 26818879

[B69] Wacongne C, Labyt E, Wassenhove V van, Bekinschtein T, Naccache L, Dehaene S (2011) Evidence for a hierarchy of predictions and prediction errors in human cortex. Proc Natl Acad Sci U S A 108:20754–20759. 10.1073/pnas.1117807108 22147913 PMC3251061

[B70] Wightman FL, Kistler DJ (1996) Individual differences in human sound localization behavior. J Acoust Soc Am 99:2470. 10.1121/1.415531

[B71] Wilson RC, Nassar MR, Gold JI (2013) A mixture of delta-rules approximation to Bayesian inference in change-point problems. PLoS Comput Biol 9:e1003150. 10.1371/journal.pcbi.1003150 23935472 PMC3723502

[B72] Winkler I, Paavilainen P, Alho K, Reinikainen K, Sams M, Näätänen R (1990) The effect of small variation of the frequent auditory stimulus on the event-related brain potential to the infrequent stimulus. Psychophysiology 27:228–235. 10.1111/j.1469-8986.1990.tb00374.x 2247552

